# Impact of supervised aerobic exercise training on habitual physical activity in healthy older adults: the Hertfordshire physical activity randomised controlled trial

**DOI:** 10.1136/bmjsem-2023-001857

**Published:** 2025-03-25

**Authors:** Francis Martin Finucane, Kate Westgate, Stephen Sharp, S J Griffin, Martin O’Donnell, Elaine Dennison, Cyrus Cooper, Nick Wareham, Soren Brage

**Affiliations:** 1 MRC Epidemiology Unit, University of Cambridge School of Clinical Medicine, Cambridge, UK; 2 Department of Medicine, University of Galway College of Medicine Nursing and Health Sciences, Galway, Ireland; 3 Cúram, University of Galway, Galway, Ireland, Ireland; 4 MRC Lifecourse Epidemiology Unit, University of Southampton, Southampton, UK

**Keywords:** Epidemiology, Physiology, Aerobic fitness, Accelerometer, Randomised controlled trial

## Abstract

**Objectives:**

Physical activity is important for health, but the influence of structured, supervised aerobic exercise sessions on habitual physical activity in healthy older adults is unclear.

**Methods:**

We evaluated habitual physical activity in the Hertfordshire Physical Activity Trial, where healthy older adults were randomised to 36 supervised 1-hour gymnasium sessions on a cycle ergometer at moderate intensity over 12 weeks or to a control group with no intervention. We estimated physical activity energy expenditure (PAEE) and time spent in sedentary behaviour and light and moderate or vigorous physical activity over 7 days at three time points (before, during and immediately after the intervention) with individually calibrated combined heart rate and movement sensing.

**Results:**

Of 100 randomised participants (44% female, aged 67–76 years), 96% completed follow-up. Midway through the intervention, neither overall PAEE nor time spent at different intensities were different between groups. However, on the 3 days of the week that the structured exercise sessions occurred (Monday, Wednesday, Friday), the exercise group had a 9.1 kJ kg^-1^ day^-1^ ((2.5, 15.7), p=0.007) increase in PAEE, a reduction in sedentary time and increased time spent at light and moderate or vigorous physical activity, compared with the control group.

**Conclusions:**

Three 1-hour bouts per week of structured aerobic exercise increased daily physical activity on the days they occurred, but not overall physical activity across the whole week. Population-wide strategies such as better cycling and walking infrastructure may increase physical activity in healthy older adults more effectively than treatment with structured exercise programmes.

**Trial registration number:**

ISRCTN60986572.

**WHAT IS ALREADY KNOWN ON THIS TOPIC**

- Physical activity is an important determinant of metabolic and cardiovascular health.
- The influence of structured, supervised aerobic exercise programmes on overall physical activity is not well described.

**WHAT THIS STUDY ADDS**

- In healthy older adults, three supervised one-hour sessions of moderate intensity aerobic exercise per week increased objectively measured physical activity, but only on the days the supervised exercise bouts occurred, with no overall effect on physical activity across the whole week.
- 180 min per week of supervised, structured, moderate intensity aerobic exercise may be insufficient to impact meaningfully on overall weekly physical activity in healthy older adults.

**HOW THIS STUDY MIGHT AFFECT RESEARCH, PRACTICE OR POLICY**

- Rather than defining lower-risk population subgroups for treatment with structured exercise programmes, it may be more impactful and efficient to adopt population-wide strategies to facilitate more active lifestyles, such as through better cycling and walking infrastructure.

## Background

 The importance of physical activity as a determinant of non-communicable disease risk is well established.^1^ High levels of physical inactivity adversely affect physical, psychological and social components of successful ageing^2^ and are an important modifiable risk factor for dementia in older adults.^3^ While increasing physical activity in older adults is an important public health strategy, uncertainty exists around the best way to achieve this. Structured exercise programmes have long been known to improve morbidity and mortality in older patients with cardiovascular disease,^4^ impaired glucose metabolism^5^ and a range of other disorders.^6^ However, whether supervised, structured exercise programmes lead to increases in habitual physical activity is less clear. Evidence from randomised controlled trials that individual-level physical activity interventions make healthy adults more active appears strong,^7^ but physical activity outcomes in these trials are usually self-reported and thus prone to bias and sometimes have unproven validity or reliability.^8^ A recent scoping review described a modest level of evidence that structured exercise interventions increase physical activity in older adults, with insufficient individual intervention studies conducted to date.^9^ A recent large multicentre randomised controlled trial in mobility-impaired older adults found an attenuation in the overall decline in physical activity over 2 years during an individually tailored combined aerobic and resistance exercise training programme.^10^ However, this intervention involved six home- and group-based exercise sessions per week. Whether fewer sessions per week or an aerobic-only exercise intervention could elicit a similar effect on physical activity has not previously been evaluated, and it is also uncertain whether these findings apply to healthy older adults.

An early study of the influence of structured exercise programmes on objectively measured physical activity in healthy older adults suggested that a reduction in physical activity energy expenditure (PAEE) might occur outside of the times that the intervention took place, such that there was no overall change in PAEE or in total energy expenditure, quantified with doubly labelled water.^11^ This ‘compensatory’ reduction in free-living energy expenditure was subsequently confirmed in healthy older adults undergoing supervised bouts of aerobic exercise with 24-hour chamber calorimetry studies^12^ and with accelerometry-based estimates of PAEE, but importantly these studies did not have control groups for comparison.^13 14^ A systematic review of 31 exercise intervention studies found minimal evidence for compensatory reductions in physical activity outside of exercise interventions: studies that did find a compensatory effect had design limitations such as no control group, whereas well-designed randomised controlled trials have not found a compensatory effect on physical activity from structured exercise.^15^

Addressing uncertainty around the influence of structured exercise programmes on habitual physical activity may help to make these programmes more effective for improving health. In this study, we aimed to determine whether a fully supervised and monitored 12 week aerobic exercise intervention in healthy older adults resulted in changes in objectively measured PAEE during the exercise intervention, compared with a control group with no intervention. We also investigated whether differences in mean daily PAEE were apparent on training days (Monday, Wednesday, Friday) compared with non-training days (Tuesday, Thursday, Saturday, Sunday) in the intervention group compared with the control group. Finally, we determined whether changes in measures of habitual physical activity in the period immediately after completion of the intervention were different in the exercise vs the control group.

## Methods

### Study design and setting

The study rationale and design^16^ and main trial findings^17^ have been described in detail previously. Briefly, in this single centre, randomised controlled trial, participants were allocated to the exercise or control group using the minimisation method, with the minimisation factors being birth weight, percentage body fat, sex and whether or not a muscle biopsy was performed at baseline. Randomisation was conducted using the sealedenvelope.com web-based randomisation service. All participants provided written, informed consent. The duration of follow-up was 12 weeks from study entry. All study measures were conducted at the MRC Epidemiology Unit, Institute of Metabolic Science in Cambridge, UK, between 17 January 2007 and 15 February 2008. The research assistants taking the measurements were unaware of the participants’ group allocations. The supervised aerobic exercise intervention was delivered at a gymnasium in Hitchin, Hertfordshire. The study protocol was approved by the Hertfordshire Research Ethics Committee (Local Research Ethics Committee ref. 05/Q0201/23).

### Study population

**Figure 1:**
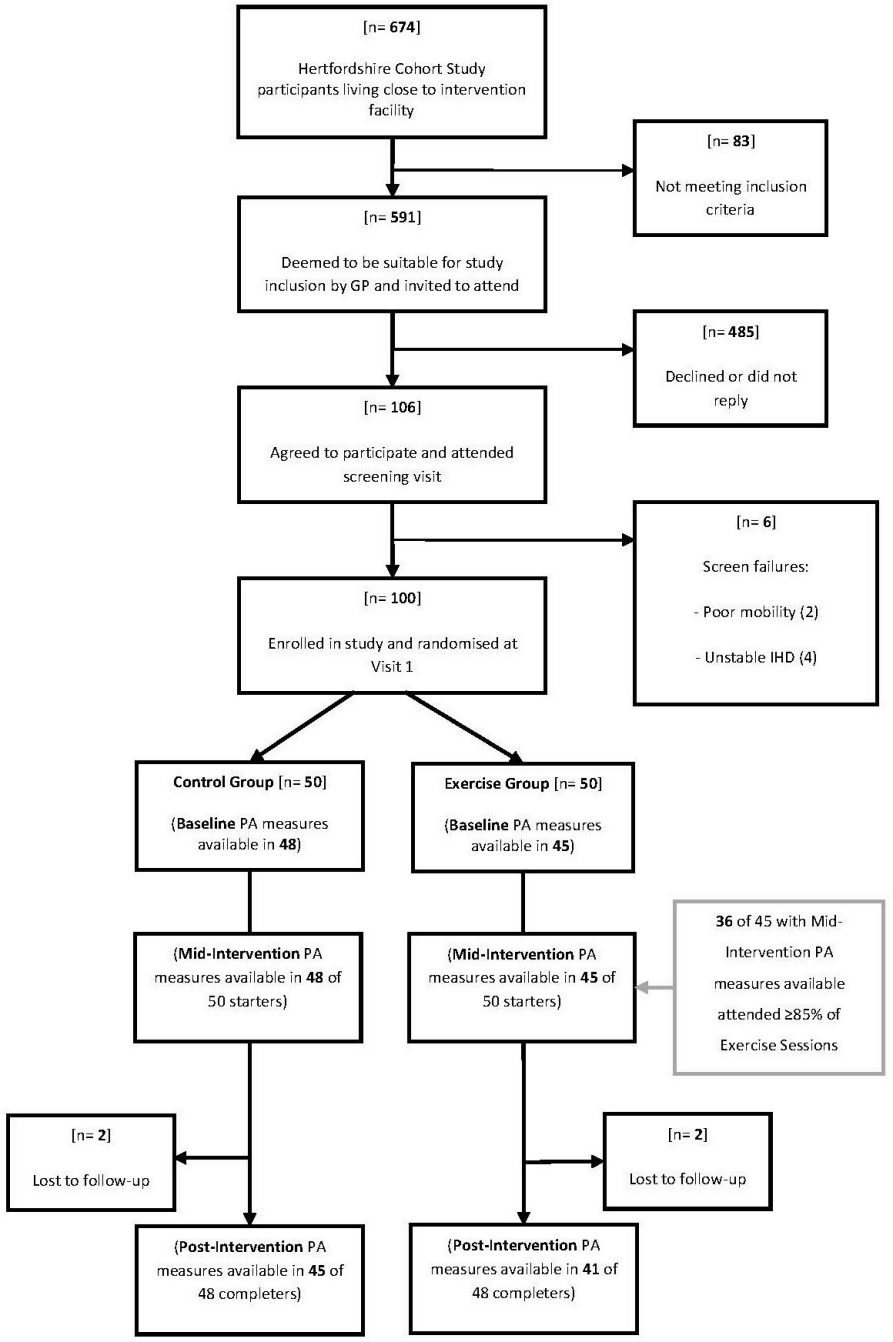
Consolidated Standards of Reporting Trials flow diagram for Hertfordshire Physical Activity Trial Recruitment.

For the original trial, we had aimed to recruit 100 participants from the Hertfordshire Cohort Study, a unique data resource consisting of almost 3000 men and women born between 1931 and 1939 and still residing in Hertfordshire.^18^ This sample size was based on a calculation for our original trial^17^ which had 85% power to detect a difference in the composite metabolic risk score (zMS) of 0.6 SD units between groups at follow-up, with the assumption that the risk score at follow-up in the control group would have a mean of 0 and an SD of 1. This original estimate of effect size was consistent with our observations in a similar population-based cohort,^19^ but the actual difference we observed in zMS between the exercise and control groups was just 0.07 (95% CI −0.17 to 0.03).^17^ Specifically, potential study participants meeting inclusion criteria, living near Hitchin and deemed potentially suitable for participation by their general practitioner were identified. We excluded those with known diabetes, untreated or unstable ischaemic heart disease or any medical condition that would prevent them from cycling unaided for at least 30 min. Invitations were dispatched on a phased basis until the recruitment target was achieved. Participants attended the clinical research facility at the Institute of Metabolic Science after an overnight fast. From the Hertfordshire Cohort Study population, we identified 674 who lived near the gymnasium in Hitchin where the intervention was to be delivered, and who did not have type two diabetes, as outlined in the Consolidated Standards of Reporting Trials flow diagram (figure 1). Of these, 83 individuals did not meet inclusion criteria, or their GP thought that study participation would not be appropriate. We invited a total of 591 individuals from the cohort, of whom 485 declined or did not respond. Of 106 who attended the screening visit, six were deemed to be unsuitable for the study because of poor mobility, diabetes, symptoms or signs suggestive of untreated ischaemic heart disease or a combination of these factors. These six were excluded.

### Patient and public involvement

Neither patients nor members of the public were involved in any aspect of the design or conception of this study. However, after completion of the trial in 2009, all participants were invited to a meeting where different members of the research team described the results and implications of the research and thanked participants for their contribution to the work.

### Equity, diversity, and inclusion statement

As the participant population was recruited from a birth cohort of individuals born between 1931 and 1939 in Hertfordshire, UK, the ethnicity of the cohort is limited exclusively to White British individuals. This limits the generalisability of the findings to other ethnic groups, which we have acknowledged in the discussion. The author team has a 2:7 ratio of women to men.

### Behavioural outcome measures of physical activity

In order to estimate average daily physical activity, all participants wore a combined heart rate and movement sensor (Actiheart, CamNtech Ltd., Papworth, UK)^20^ for 1 week prior to the start of the exercise programme, then again at the start of the 6th week of the programme and for a third time during the week immediately after completion of the programme. The control group participants wore the device for the equivalent three periods. It was applied to the upper part of the chest using standard ECG electrodes. The central component was placed at the level of the left third intercostal space, with a 100 mm wire running horizontally to the second smaller component, placed medial to the left axillary fold. A 2-min signal test was then conducted to ensure correct electrode placement and detection of cardiac electrical activity. Participants then underwent a submaximal cycle ergometer test as described previously,^16^ allowing individual calibration of heart rate to physical activity intensity. The test had a starting workload of 50 W, increasing by 10 W every min until 90% of the maximum age-predicted heart rate was achieved or the participant wanted to stop, whichever came sooner. We obtained an estimate of maximal aerobic fitness by extrapolating a regression line between the observed heart rate and the obtained maximum power output (predicted) (W_max_) up to the age-predicted maximal heart rate.^21^ The sensing device was removed, and its data were downloaded and then reinitialised and replaced on the anterior chest wall for 7 days, continuously recording free-living heart rate and acceleration in 15-s epochs. Participants were provided with an instruction sheet and replacement ECG electrodes and were asked to record time periods when the device was removed and reasons for removal. They were encouraged to continue with their usual activities and to wear the monitor at all times, whether awake or asleep. Devices were returned by prepaid registered post after 7 days, and the raw data were immediately downloaded.

These data were extracted and cleaned in two phases. The first involved identifying and removing outlier values using fixed-cluster means, and the second involved utilising a Gaussian regression process to infer latent time series where data were missing.^22^ The raw data and the inferred heart rate were then plotted together with wear/non-wear classification (inferred from prolonged periods of non-physiological heart rate and no acceleration) and manually reviewed. Instantaneous PAEE, or activity intensity (J kg^-1^ min^-1^) for each time point, was estimated from the combination of movement registration and individually calibrated heart rate using a branched equation framework.^23 24^ We have recently described validation of this approach, comparing measured vs estimated energy costs of treadmill exercise in UK adults.^25^ Periods of non-wear were taken into account to minimise diurnal information bias when summarising the intensity time series into overall volume of daily PAEE (kJ kg^-1^ day^-1^). (For the mid-intervention measurements of physical activity, the mean of the individual calibration factors from the baseline and follow-up study visits was applied.) If the calibration factor for an individual was not available at either baseline or follow-up visits, their usable visit was scaled for the missing time-point. This approach to quantifying PAEE has previously been successfully validated by our group against indirect calorimetry during simulated daily living activities^26^,^28^ and during free-living against doubly labelled water.^29^ In these studies, treadmill-calibrated estimates agreed with isotope measures and correlated strongly (r=0.67), regardless of using or not using individual-level measurement of respiratory gas exchange from the treadmill test. Step- and walk-test calibrated results performed similarly, but non-exercise calibrated models were less precise. Across all of these estimates, the accelerometer model remains the same, emphasising the importance of individually calibrating heart rate to energy expenditure, even when it is used in combination with accelerometry. Previous studies have suggested consistency and universality in the relationship between individual heart rate and energy expenditure across different activities, as long as the large muscle groups of the legs are engaged.^30^ In the present study, we calibrated the heart rate to energy relationship using leg cycle ergometry and combined that signal with the non-individualised Newtonian accelerometry model for rest-walking-running intensity.

### Other measurements

All measurements were undertaken by trained staff adhering to standard operating procedures. Weight was measured on a Tanita (Tokyo, Japan) scale and height with a Seca (Hamburg, Germany) rigid wall-mounted stadiometer. Waist and hip circumferences were measured using a D-loop non-stretch fibreglass tape measure. The waist was defined as the midpoint between the lower costal margin and the level of the superior iliac crests. The hip measurement was taken at the level of the greater trochanter. A questionnaire was used to record details of current medications, smoking and alcohol use. All of these measurements were repeated 48 hours after completion of the exercise programme.

### Exercise intervention

Participants attended the exercise facility for three 1-hour sessions per week over 12 weeks, on Monday, Wednesday and Friday mornings or afternoons. All sessions were fully supervised by the exercise facilitator and incorporated a warm-up and cool-down period of 5 min each. Participants used upright or recumbent cycle ergometers (according to their preference) to achieve an exercise intensity of 50, 60 and 70% of W_max_ during weeks 1 to 4, 5 to 8 and 9 to 12 of the intervention, respectively. To ensure that the appropriate intensity was maintained during the sessions, heart rate was monitored (Polar F4 monitors; Bodycare Products, Southam, UK) and recorded at 5-min intervals. For participants on beta-blockers, the Borg scale^31^ was used to assess the intensity of exercise. For those randomised to the exercise intervention, a follow-up visit to the testing facility was scheduled for precisely 2 days after completion of the final exercise session. Participants randomised to the control group were asked to continue with their usual levels of physical activity until their follow-up visit 12 weeks later.

### Statistical methods

In order to determine whether the 12-week supervised aerobic exercise programme was associated with changes in mean daily PAEE over 7 days, we compared differences in the changes between the exercise and control groups. Specifically, in linear regression analyses, we modelled the mid-intervention measure (at week 6) of mean daily PAEE over 7 days as the outcome variable and the randomisation group as the exposure variable, adjusting for the pre-intervention measure of PAEE. We included all participants in the group to which they were randomised and also performed a secondary ‘per protocol’ analysis, comparing the control group with participants who attended 85% or more of the 36 exercise sessions. We used a similar approach for the other objectively measured physical activity outcome variables, specifically the proportion of time spent under 1.5 standard metabolic equivalents (METs) (ie, sedentary behaviour), between 1.5 and 3 METs (light physical activity) and above 3.0 METs (moderate or vigorous physical activity (MVPA)). When we compared differences between exercise and control participants specifically on the days of the week corresponding to when the intervention was delivered (Monday, Wednesday, Friday) and then again on the days corresponding to when the intervention was not delivered (Tuesday, Thursday, Saturday, Sunday), we used linear regression with a random intercept to allow for the repeated measurements on each individual. Missing physical activity measures at baseline or follow-up were excluded from the analysis. Specifically, participants were excluded from the analyses if they had not worn their sensor for at least 72 hours overall and for at least 15 hours of cumulative wear for each quadrant of the day (00:00–06:00, 06:00–12:00, 12:00–18:00, 18:00–00:00). These latter criteria ensured that behavioural information was available for all parts of the day, from at least three different days. For the comparison of days when the intervention was delivered compared with those when it was not delivered, participant days were excluded if the participants had not worn their sensor for at least 12 hours that day and with no quadrant requirement. As a sensitivity analysis, we repeated this day-level comparison with a wear criterion of at least 16 hours in total and 2 hours in each quadrant.

## Results

Of the 100 participants who attended visit one and were randomised, 50 were randomised to each intervention group. Two participants in each group dropped out of the study, and for reasons not related to the study, they declined to attend for their follow-up visit. Among participants randomised to the exercise group, 37 (74%) attended 85% or more of their scheduled exercise sessions, while 20% attended all scheduled sessions. Five participants in the exercise group attended fewer than one third of their scheduled exercise sessions, but still returned for follow-up measures. Of these, three never started the intervention, one stopped after 4 weeks due to exacerbation of preexisting osteoarthritis in both knees and one stopped after 2 weeks because of exercise-induced palpitations, although subsequent cardiac investigations in this participant were normal. Physical activity measures were available in 93 of 100 participants at baseline and midway through the intervention, with inadequate duration of sensor wearing for inclusion in seven participants. Postintervention physical activity measures were available in 86 of 96 participants who attended for their follow-up visit, with inadequate duration of sensor wearing for inclusion in 10 participants. Of the 37 exercise participants who attended 85% or more of their prescribed exercise sessions, 36 had mid-intervention physical activity measures available (with one excluded because of inadequate duration of sensor wearing), as shown in figure 1. Participants in the exercise and control groups had similar baseline characteristics, as shown in table 1.

**Table 1:** Baseline characteristics of Hertfordshire Physical Activity Trial participants

Variable	Control group	Exercise group
n	48	45
Age (years)	71.5±2.7	71.3±2.2
Sex		
Female, n (%)	22 (45.8%)	19 (42.2%)
Male, n (%)	26 (54.2%)	26 (57.8%)
Smoking status		
Current smoker (%)	0 (0%)	2 (4.4%)
Ex-smoker (%)	19 (39.6%)	15 (33.3%)
Never smoker (%)	29 (60.4%)	28 (62.2%)
Alcohol (units per week)*	2 (1, 8)	4 (1, 10)
Glucose tolerance status		
Normal glucose tolerance (%)	30 (62.5%)	26 (57.8%)
Impaired glucose metabolism (%)	14 (29.2%)	18 (40%)
Incident type 2 diabetes (%)	4 (8.3%)	1 (2.2%)
Metabolic syndrome		
Yes (%)	16 (33.3%)	13 (28.9%)
No (%)	32 (66.7%)	32 (71.1%)
Medication usage		
ACE inhibitor/ARB (%)	11 (22.9%)	11 (24.4%)
Calcium channel blocker	7 (14.6%)	8 (17.8%)
Beta-blocker (%)	7 (14.6%)	7 (15.6%)
Diuretic (%)	5 (10.4%)	6 (13.3%)
Statin (%)	11 (22.9%)	8 (17.8%)
Weight (kg)*	77.9 (66, 84.9)	71.8 (64.7, 87.6)
Height (cm)	169.1±9.6	167.5±7.8
BMI (kg m^-2^)*	26.7 (24.1, 28.7)	26.4 (24, 29.7)
Systolic blood pressure (mm Hg)	134.3±17.5	138±15.2
Diastolic blood pressure (mm Hg)	73.3±9	76.1±8.5
Predicted maximal watts (W)*	139.2 (112.1, 207.5)	135.8 (109.5, 173.3)
Physical activity measure:		
Overall physical activity kJj^-1^ kg^-1^ day^-1^)*	33.3 (24.7, 37.5)	30.5 (20.2, 38.4)
Overall physical activity (kJ^-1^ kg^-1^ day^-1^)†	23.6±6.8	23.3±9.2
Percentage time under 1.5 METs (%)	77.6±7.8	78±10
Percentage time 1.5 to 3 METs (%)	20.1±7.1	19.7±8.3
Percentage time above 3 METs (%)*	1.6 (0.9, 2.8)	1.9 (0.5, 3.3)
Actual time under 1.5 METs (mins/day)	1117.4±112.3	1123.2±144
Actual time 1.5 to 3 METs (mins/day)	289.4±102.2	283.7±119.5
Actual time above 3 METs (mins/day)*	23 (13, 40.3)	27.4 (7.2, 47.5)

* Continuous variables with a symmetric distribution are summarised using mean ± standard deviation, those with a skewed distribution (denoted *) are summarised using median (interquartile range).

† Denotes overall physical activity, measured with accelerometry only, rather than combined heart rate and movement sensing.

ACE, angiotensin converting enzyme; ARB, angiotensin receptor blocker; BMIbody mass indexMET, Metabolic Equivalent of Task

### Changes during the exercise intervention

There was no evidence of change during the intervention in mean daily PAEE over 7 days in the exercise group compared with the control group in linear regression analyses adjusted only for the baseline measure of the outcome variable, as shown in table 2. Results were similar in per-protocol analyses, including only those 37 participants in the exercise group who completed ≥85% of the prescribed sessions (as shown in online supplemental table 1). Furthermore, there was no evidence of changes in the proportion of time spent under 1.5 METs (sedentary behaviour), between 1.5 and 3 METs (light physical activity) or over 3 METs (MVPA), in the exercise group compared with the control group during the intervention.

**Table 2:** Physical activity–related measures in exercise and control groups before, during and after the Hertfordshire Physical Activity Trial

Variable	Control group			Exercise group			Difference in change during intervention†	P value	Difference in change after intervention ‡	P value
	**Pre**	**Mid**	**Post**	**Pre**	**Mid**	**Post**				
N	48	48	45	45	45	41				
Overall physical activity										
PAEE(kj^-1^ kg^-1^ day^-1^)*	33.3 (24.7, 37.5)	32.9 (23.9, 41)	31.2 (22.4, 43.5)	30.5 (20.2, 38.4)	31.9 (24.1, 43.1)	31.8 (27, 39.4)	0.1 (−3.2, 3.4)	0.95	0.2(-3.9, 4.3)	0.93
PAEE (accelerometry only)(kj^-1^ kg^-1^ day^-1^)	23.6±6.8	23.9±8.2	22.4±9.8	23.3±9.2	23.6±9.4	23.3±9.9	−0.1 (−2.8, 2.7)	0.96	1.1(-2.2, 4.4)	0.5
Percentage time <1.5 METs (%)(sedentary behaviour)	77.6±7.8	77±7.8	77.4±8.6	78±10	76.2±8.6	75.5±8.8	−1.1 (−3.3, 1.1)	0.31	−2.2(-4.8, 0.4)	0.093
Percentage time 1.5 to 3 METs (%)(light physical activity)	20.1±7.1	20.4±6.9	19.5±7.2	19.7±8.3	21.4±7.5	22±7.7	1.3 (−0.7, 3.2)	0.21	2.9(0.7, 5.1)	0.012
Percentage time >3 METs (%)*(moderate to vigorous PA)	1.6 (0.9, 2.8)	2 (0.8, 3.7)	2.1 (0.8, 4.5)	1.9 (0.5, 3.3)	1.9 (0.9, 3.6)	1.8 (0.9, 3.5)	−0.1 (−0.8, 0.6)	0.68	−0.7(-1.5, 0.1)	0.085
Actual time <1.5 METs (mins/day)(sedentary behaviour)	1117.4±112.3	1108.8±112.3	1114.6±123.8	1123.2±144	1097.3±123.8	1087.2±126.7	−15.8 (−47.5, 15.8)	0.31	−31.7(-69.1, 5.8)	0.093
Actual time 1.5–3 METs (mins/day)(light physical activity)	289.4±102.2	293.8±99.4	280.8±103.7	283.7±119.5	308.2±108	316.8±110.9	18.7 (−10.1, 46.1)	0.21	41.8(10.1, 73.4)	0.012
Actual time >3 METs (mins/day)*(moderate to vigorous PA)	23 (13, 40.3)	28.8 (11.5, 53.3)	30.2 (11.5, 64.8)	27.4 (7.2, 47.5)	27.4 (13, 51.8)	25.9 (13, 50.4)	−1.4 (−11.5, 8.6)	0.68	−10.1(-21.6, 1.4)	0.085

Results were also similar in per-protocol analyses, where only the 37 participants who completed≥85% of the prescribed sessions were considered in the exercise group.

* Denotes variables with a skewed distribution, in which case the median and (interquartile range) are presented. Otherwise, data are presented as means ± standard deviations.

† Refers to the difference in the change in the outcome during the intervention in the exercise group compared to the control group, using linear regression with the outcome variable being the mid-intervention follow-up measure at week six, and the exposure variable being the randomisation group, adjusted for the baseline measure of the outcome.

‡ Refers to the difference in the change in the outcome after the intervention in the exercise group compared to the control group, using linear regression with the outcome variable being the post-intervention follow-up measure, and the exposure variable being the randomisation group, adjusted for the baseline measure of the outcome.

MET, metabolic equivalent of taskPAEEphysical activity energy expenditure

**Figure 2:**
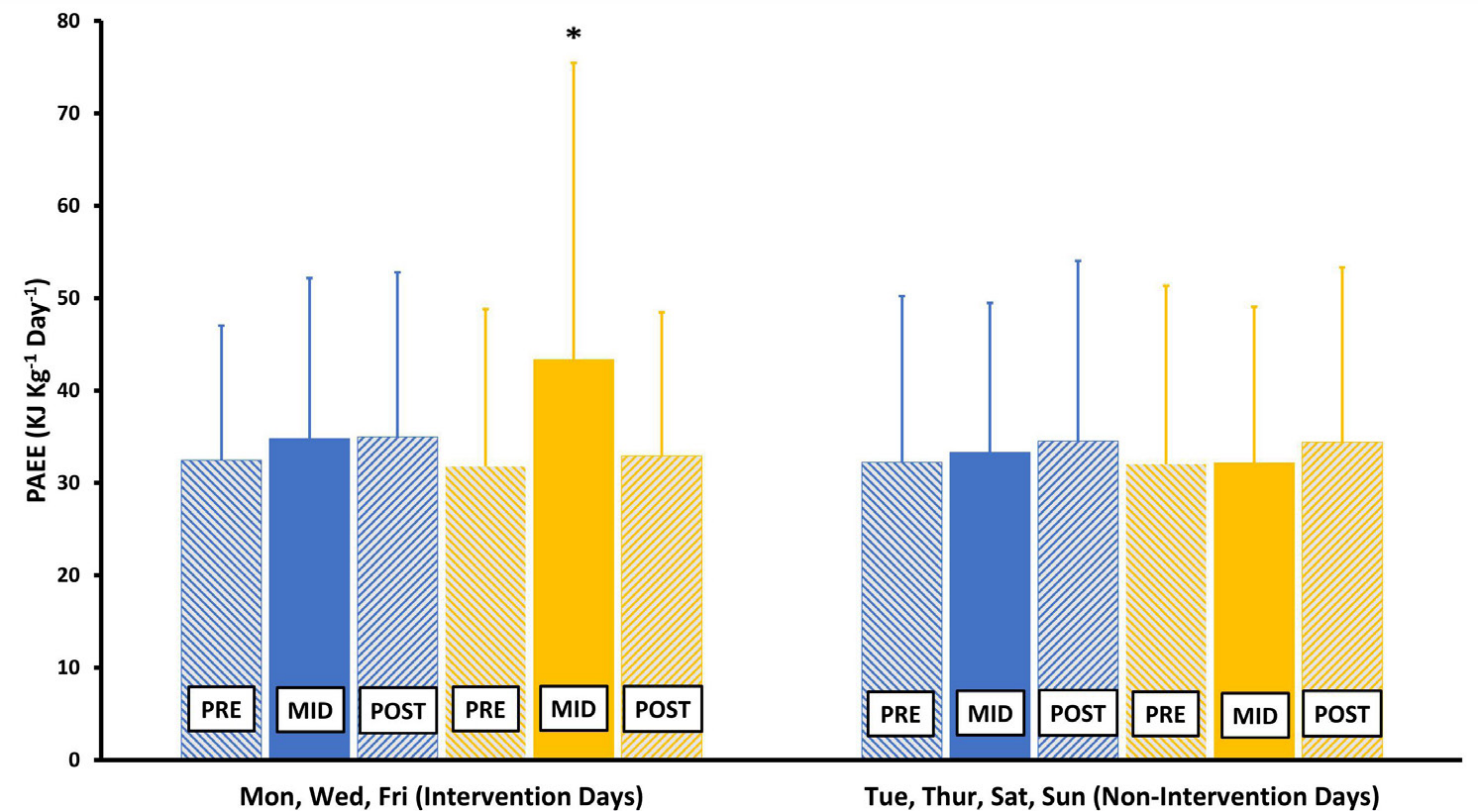
Physical activity energy expenditure before, during and after completion of the 12-week supervised, structured aerobic exercise intervention on Monday, Wednesday and Friday (exercise intervention days) compared with Tuesday, Thursday, Saturday and Sunday (non-exercise intervention days). Comparisons of PAEE before, during and after the exercise intervention were made using multilevel mixed-effects linear regression analyses and refer to the difference in follow-up PAEE, adjusted for baseline PAEE, according to trial randomisation group. *p=0.007 for difference in mid-intervention PAEE on exercise intervention days. No other comparisons were statistically significantly different.

**Figure 3:**
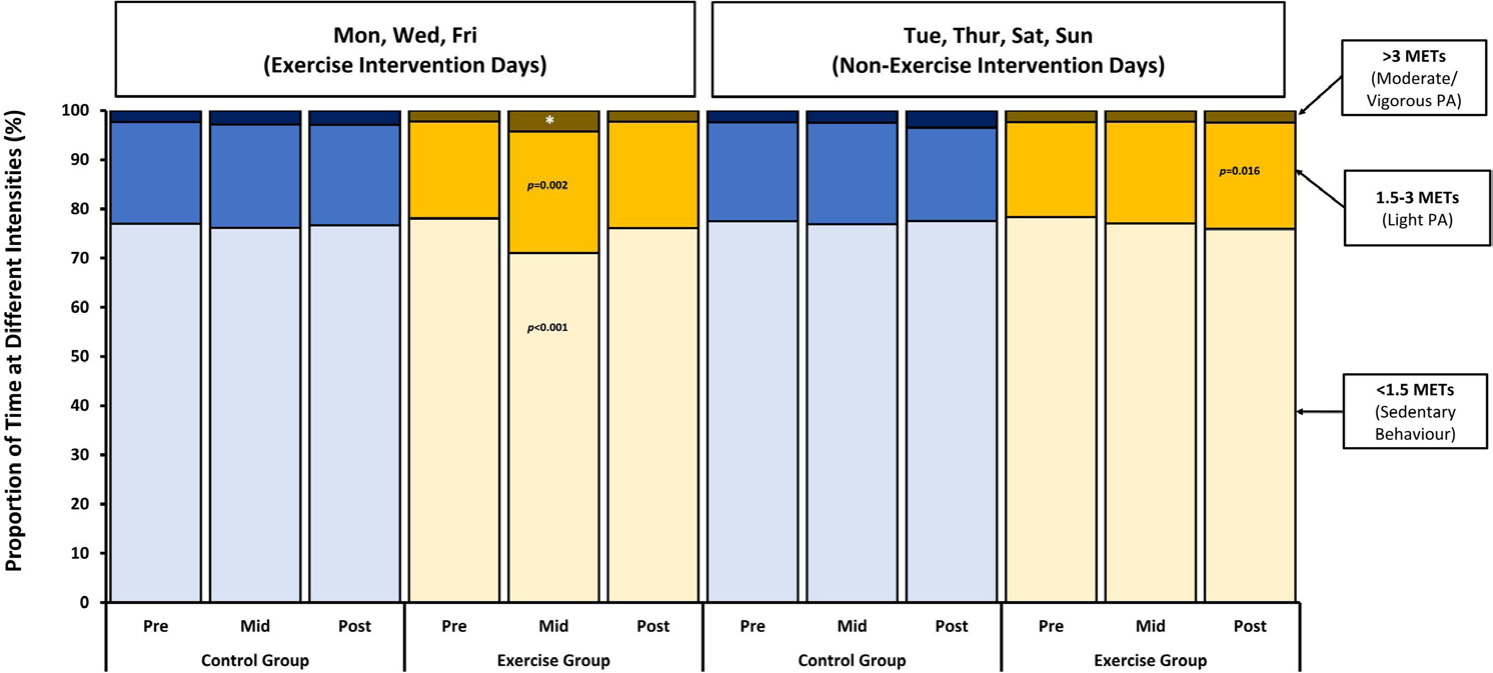
Distribution of mean proportions of time spent at different levels of physical activity intensity before, during and after completion of the 12-week supervised, structured aerobic exercise intervention on Monday, Wednesday and Friday (exercise intervention days) compared with Tuesday, Thursday, Saturday and Sunday (non-exercise intervention days). Linear regression was used to compare the change in the proportion of time at each physical activity intensity level midway through the intervention in the exercise group compared with the control group, adjusted for the baseline proportion of time at that physical activity intensity level. *Denotes p=0.042. None of the other changes in proportions of time at different physical activity intensity levels were statistically significant. MET, metabolic equivalent of task.

To investigate whether the exercise intervention had any effect on physical activity outcomes on the specific days that the supervised gym sessions took place, we repeated all of the analyses outlined above separately for the specific days of the week that the exercise intervention was delivered (Monday, Wednesday, Friday) and for the days that the exercise intervention was not delivered (Tuesday, Thursday, Saturday, Sunday). On the specific days of the week corresponding to when the exercise intervention was delivered, there were statistically significant increases in PAEE, as shown in figure 2 and online supplemental table 2. We also noted reductions in the proportion of time spent in sedentary behaviour under 1.5 METs and increases in the proportion of time spent in light physical activity between 1.5 and 3 METs and in MVPA above 3 METs on the days that the exercise intervention took place in the exercise group compared with the equivalent days in the control group, as shown in figure 3 and online supplemental table 2. On the days corresponding to exercise intervention delivery, the proportion of time spent under 1.5 METs was 5.6% (2.5, 8.6) lower (p<0.001), (equivalent to 80.6 (36, 123.8) fewer minutes of sedentary behaviour per day), while the proportion of time spent between 1.5 and 3 METs was 4% (1.4, 6.6) higher (p=0.002) (equivalent to 57.6 (20.2, 95) min more of light physical activity per day), and the proportion of time spent above 3 METs was 1.6% (0.1, 3.1) higher (p=0.042) (equivalent to 23 (1.4, 44.6) min more of MVPA per day) compared with the control group. However, there were no differences in any of these outcomes between the exercise and control groups on the days corresponding to when the exercise intervention was not delivered. In a sensitivity analysis where we applied more stringent minimal wear time criteria of 16 hours per day and 2 hours in each quadrant, the number of participant-days with physical activity outcomes available dropped from 587 to 471, and the changes in physical activity outcomes in the exercise group compared with the control group on exercise intervention days were no longer apparent, as shown in online supplemental table 3.

### Changes after the exercise intervention

There was no evidence of between-group differences in the magnitude of changes in any week-long physical activity measures after completion of the intervention (compared with the start of the intervention), except for an increase in the proportion of time between 1.5 and 3 METs in the exercise group participants compared with the control group of 2.9 (0.7, 5.1) % (p=0.012; equivalent to 41.8 (10.1, 73.4) min of light physical activity per day), as shown in table 2. The observed increase in the overall proportion of time spent in light physical activity between 1.5 and 3 METs seen in the exercise group participants after completion of the intervention was only apparent on the days corresponding to when the exercise intervention had not been delivered, not to the days when it was delivered, as shown in figure 3 and online supplemental table 1.

## Discussion

In this randomised controlled trial in healthy older adults, we found no evidence that a structured exercise intervention involving three 1-hour sessions per week was associated with changes in overall physical activity over the week. We were surprised that the structured exercise intervention had no influence on any of the week-long physical activity outcomes, even after including only participants who adhered to the protocol, particularly as we have already described how this trial found improvements in fitness and some cardiovascular risk markers in the intervention group.^17^ Our study is the first randomised controlled trial to describe the effects of a supervised, structured aerobic exercise programme on PAEE and related measures of physical activity intensity distribution in healthy older adults. Our findings are important because they suggest that the frequency (as opposed to the intensity, modality or duration) of the exercise sessions was not adequate to influence average physical activity for the week. The fact that we were able to detect a difference in PAEE between the intervention and control group participants on the days that the supervised exercise bouts occurred suggests that we have adequate sensitivity to see a difference when it is there. It makes it less likely that our ‘null’ overall trial findings have arisen from imprecision or error in the measurement of the physical activity variables. We think that if there was inaccuracy or imprecision, they would introduce random error, rather than bias, and that the net result would be inflated variance of the estimates of PAEE, which could indeed mask small true differences. This is reflected in the observation that with more stringent wear time criteria, 116 of 587 participant days were removed from the analysis, and changes in physical activity outcomes were no longer apparent or statistically significant on exercise intervention days in the intervention group compared with the control group. The effect of structured exercise on habitual physical activity on the specific days the intervention was delivered was consistent across all of the physical activity outcomes. Our observation of an increase in the time spent in light physical activity after completion of the intervention in the exercise group, but only on the days corresponding to when the exercise intervention was not delivered, may have arisen by chance with multiple statistical comparisons or may reflect a true effect of the intervention.

The observation of higher physical activity in the exercise group on the specific exercise intervention days but not over the whole week suggests that there may have been a compensatory reduction in physical activity in the exercise group on the days that the intervention was not delivered, but we have not demonstrated such a reduction here. A previous study describing compensatory reductions in free-living energy expenditure with supervised bouts of aerobic exercise in healthy older adults^12^ used a physical activity questionnaire combined with chamber calorimetry to quantify energy expenditure, but had no control group. Others have described compensatory reductions in ‘non-training’ physical activity, quantified with accelerometry, during supervised, structured exercise programmes, but again without a control group for comparison.^13 14^ A recent trial of the effects of 2 weeks of supervised exercise on accelerometry-derived physical activity measures in overweight young adult male naval recruits in Brazil noted a compensatory reduction in physical activity in the exercise intervention group.^32^ Others have proposed that increased exercise does not necessarily change total daily energy expenditure (TDEE), because of compensatory changes in either resting energy expenditure or in non-exercise PAEE.^33^ An alternative model of TDEE proposes that compensatory reductions in energy expenditure might extend to diet-induced thermogenesis and other less well-defined domains of energy expenditure.^34^ We did not measure resting energy expenditure in our study and can therefore not comment on possible changes in this component. While our results do not provide conclusive proof of the phenomenon of compensatory reduction in PAEE outside of an exercise training programme, this warrants exploration in future studies.

Given that exercise training can reduce heart rate for a given task, we considered the possibility that this might influence estimates of physical activity from combined heart rate and movement sensing. We adopted two strategies to mitigate this limitation. First, we incorporated a repeat exercise test for individual calibration of heart rate to energy expenditure at the end of the study. Though we did not do this midway through the study, we used the average calibration between baseline and follow-up to calibrate the midpoint measurements. This strategy takes account of the influence of improved fitness and reduced heart rate on estimates of physical activity. Therefore, we do not think that reduced heart rate would account for the absence of an overall effect of the exercise intervention on physical activity. Second, when we derived an estimate of physical activity using only accelerometry data (and not heart rate), our results were similar, as shown. We have used the same methodological approach as a previous study of 35 healthy French men of varying weight status and physical activity levels, where Actiheart-derived combined accelerometry and heart rate measures of PAEE correlated better with doubly labelled water measures of activity energy expenditure than estimates from heart rate or accelerometry alone. More importantly, this approach was adequately sensitive to detect changes in activity energy expenditure induced by an exercise intervention, particularly if the individual calibration procedure for heart rate was repeated.^35^ Of note, this study used a cycle ergometry test for individual calibration, as we have done.

Given that the benefits of structured aerobic exercise programmes are already well established in less healthy individuals,^36^,^40^ the relatively good health of this cohort is a strength of our study. Participants in our trial had relatively low levels of PAEE at baseline compared with representative average values in older UK adults.^41^ Also, all participants had a White British ethnic background, were born in the same region (Hertfordshire) between 1931–39 and still lived there at the time the trial took place. This limits the generalisability of the study to other ethnic groups, but it also minimises the potential confounding effects of ethnic and cultural factors on the response to the exercise intervention, which is a strength. Another factor limiting the generalisability of our findings is the relatively low recruitment rate of 18%. Another limitation is that while the exercise intervention was well defined, our findings are not generalisable to other aerobic exercise modalities (such as walking, running) or to resistance exercises, nor do they apply to interventions with different frequency, intensity, or duration of training or to cohorts of different age and ethnicity. In fact, more recent international physical activity guidelines emphasise the need for combined aerobic and resistance exercises to promote and maintain health, particularly in older people,^1^ whereas our intervention focused exclusively on aerobic exercise. Moreover, pragmatic, ‘real-world’ lifestyle interventions tend to combine dietary and physical activity strategies, and recent studies have confirmed the additional benefits that structured exercise brings to these interventions, compared with diet alone.^42 43^ Nonetheless, the fact that each bout of exercise was fully supervised and the level of intensity carefully defined, with continuously monitored participant exertion, has optimised fidelity of intervention delivery and minimised heterogeneity in the exercise intervention ‘exposure’. Finally, a limitation of our study design was a lack of contextual data about the types and domains of physical activity being undertaken on exercise versus non-exercise days.

## Conclusions

What are the implications of the findings in our study? First, it may be that 180 min per week of structured aerobic exercise is insufficient to impact meaningfully on overall weekly physical activity levels in healthy older adults. Put another way, if there are 168 hours in the week, the level of physical activity during the 98.2% of the time that our participants were not undergoing the supervised, structured aerobic exercise sessions is likely to have dwarfed any direct influence of those sessions, especially on the days of the week that the intervention did not take place. Second, future evaluations of structured aerobic exercise programmes should probably include a resistance training component, in line with current guidelines,^1^ and could also include advice to participants to maintain other physical activity behaviours occurring outside of the structured exercise sessions. A third implication of our study is that rather than defining specific lower-risk subgroups in the population (such as ours, without prevalent diabetes or cardiovascular disease and with good mobility) for treatment with structured exercise programmes, it may be more impactful and efficient to adopt population-wide strategies to facilitate a more active lifestyle, such as through better cycling or walking infrastructure,^44 45^ though how to provide ideal conditions for outdoor cycling in older adults remains uncertain.^46^ Making the built environment more conducive to physical activity for the whole population might be more effective than encouraging individuals to participate in structured exercise programmes, which cannot be delivered indefinitely and even during delivery, occupying a very small proportion of an individual’s time. The current bias towards short-term interventional studies in controlled settings with selected samples (such as we have described here) and away from population-based and large-scale physical activity research has already been identified as a challenge to be addressed in future studies in this field.^9^ Structured community exercise programmes still have value as a strategy to promote habitual physical activity in older adults.^47^ In higher-income countries where it is feasible to deliver such programmes, increasing their availability and access remains an important direction for future implementation studies and policy. However, it may be that for healthy (and thus relatively low risk) older adults, population-wide strategies to increase physical activity, rather than individualised treatment with structured exercise programmes, are the optimal approach for public health policy makers to take, in line with the fundamental principles of preventive medicine.^48^

## supplementary material

10.1136/bmjsem-2023-001857 online supplemental file 1

10.1136/bmjsem-2023-001857 online supplemental file 2

10.1136/bmjsem-2023-001857 online supplemental file 3

10.1136/bmjsem-2023-001857 online supplemental file 4

## Data Availability

Data are available upon reasonable request.
