# Supplementary material for: Impact of supervised aerobic exercise training on habitual physical activity in healthy older adults: the Hertfordshire physical activity randomised controlled trial
Source: BMJ Open Sport Exerc Med. 2025 Mar 25;11(1):e001857. doi: 10.1136/bmjsem-2023-001857 (PMC11938243; doi:10.1136/bmjsem-2023-001857)
Supplement: online supplemental file 1 [file bmjsem-11-1-s001.docx]

**Supplemental Table 1: Physical Activity- Related Measures in Exercise and Control Groups Before, During and After the Hertfordshire Physical Activity Trial Across the Whole Week and According to Days of the Week that the Exercise Intervention was Delivered – *Per Protocol Analyses*.**

| **Variable** | **Control Group** | | | **Per Protocol Exercise Group** | | | **Difference in Change During Intervention **** | **P-value** | **Difference in Change After Intervention ***** | **P-value** |
| --- | --- | --- | --- | --- | --- | --- | --- | --- | --- | --- |
|  | **Pre** | **Mid** | **Post** | **Pre** | **Mid** | **Post** |  |  |  |  |
| **Overall Physical Activity (Whole Week):** | | | | | | |  |  |  |  |
| N | 46 | 46 | 45 | 36 | 36 | 35 |  |  |  |  |
| PAEE  (kj^-1^ kg^-1^ day^-1^) * | 33.9 (25.5, 37.6) | 33.5 (25, 41.3) | 31.2 (22.4, 43.5) | 29.9 (20.1, 39.2) | 30.7 (23.6, 42.6) | 30.1  (26.1,  38.6) | -0.5 [-4, 3.1] | 0.79 | -1.4 [-5.6, 2.9] | 0.53 |
| PAEE (Accelerometry Only)  (kj^-1^ kg^-1^ day^-1^) | 24 ± 6.7 | 24.4 ± 8 | 22.4 ± 9.8 | 23.2 ± 9.2 | 23.7 ± 9.2 | 22.7 ± 9.3 | -0.1 [-3.1, 2.8] | 0.93 | 0.86 [-2.69, 4.41] | 0.63 |
| Percentage Time < 1.5 METs (%)  (Sedentary Behaviour) | 77.1 ± 7.5 | 76.4 ± 7.4 | 77.4 ± 8.6 | 78.1 ± 10.2 | 76.2 ± 8.3 | 76.1 ± 8.8 | -0.9 [-3.1, 1.4] | 0.45 | -1.5 [-4.1, 1.2] | 0.28 |
| Percentage Time 1.5 to 3 METs (%)  (Light Physical Activity) | 20.5 ± 6.9 | 20.8 ± 6.7 | 19.5 ± 7.2 | 19.5 ± 8.5 | 21.3 ± 7.2 | 21.6 ± 7.7 | 1.1 [-0.9, 3.1] | 0.29 | 2.3 [0, 4.6] | 0.05 |
| Percentage Time > 3 METs (%) *  (Moderate to Vigorous PA) | 1.7  (1.1, 2.8) | 2.2  (0.9, 3.8) | 2.1  (0.8, 4.5) | 1.9  (0.5, 3.5) | 1.9  (0.9, 3.6) | 1.8  (0.8, 3.5) | -0.2 [-1, 0.5] | 0.56 | -0.9 [-1.7, -0.1] | 0.039 |
| Actual Time <1.5 METs (mins/ day)  (Sedentary Behaviour) | 1110.2 ± 108 | 1100.2 ± 106.6 | 1114.6 ± 123.8 | 1124.6 ± 146.9 | 1097.3 ± 119.5 | 1095.8 ± 126.7 | -13 [-44.6, 20.2] | 0.45 | -21.6 [-59, 17.3] | 0.28 |
| Actual Time 1.5-3 METs (mins/ day)  (Light Physical Activity) | 295.2 ± 99.4 | 299.5± 96.5 | 280.8 ± 103.7 | 280.8 ± 122.4 | 306.7± 103.7 | 311 ± 110.9 | 15.8 -13, 44.6] | 0.29 | 33.1 [0, 66.2] | 0.05 |
| Actual Time > 3 METs (mins/ day) *  (Moderate to Vigorous PA) | 24.5  (15.8, 40.3) | 31.7 (13, 54.7) | 30.2 (11.5, 64.8) | 27.4  (7.2, 50.4) | 27.4  (13, 51.8) | 25.9  (11.5, 50.4) | -2.9 [-14.4, 7.2] | 0.56 | -13 [-24.5, -1.4] | 0.039 |
| **Physical Activity on Mon, Wed, Fri (Exercise Intervention Days):** | | | | | | |  |  |  |  |
| N | 128 | 125 | 120 | 93 | 93 | 83 |  |  |  |  |
| PAEE  (kj^-1^ kg^-1^ day^-1^) * | 32.1  (21.2, 40) | 32.8  (22.2, 44.6) | 30  (21.8, 47.4) | 28.6  (18.2, 42.9) | 36.9 (26.7, 51.1) | 31  (21, 42.3) | 7.6 [0.8, 14.3] | 0.029 | -1.6 [-6.9, 3.7] | 0.55 |
| PAEE (Accelerometry Only)  (kj^-1^ kg^-1^ day^-1^) * | 24.6  (17, 30) | 23.3  (17.4, 31.7) | 20.6  (15, 30) | 22  (16, 32.1) | 23.3  (17.9, 34), | 20.6  (17.2, 27.6) | 3.6 [-0.8, 8] | 0.11 | 0.5 [-3.4, 4.4] | 0.81 |
| Percentage Time < 1.5 METs (%) *  (Sedentary Behaviour) | 77.4 (70.7, 84.2) | 77 (69.3, 83.5) | 78.4 (70.4, 85.2) | 79 (69.9, 87.4) | 72.5 (64.9, 79.6) | 77.7 (68.3, 83.8) | -4.9 [-8.1, -1.7] | 0.002 | -0.9 [-4.1, 2.2] | 0.57 |
| Percentage Time 1.5 to 3 METs (%) *  (Light Physical Activity) | 19.5 (14, 26.4) | 20 (14.1, 27.4) | 19.1 (13.8, 25.9) | 18.6 (11.7, 27.9) | 23.3 (18.6, 31) | 20.8 (14.2, 27.3) | 3.7 [0.9, 6.5] | 0.009 | 1.5 [-1.3, 4.2] | 0.3 |
| Percentage Time > 3 METs (%) *  (Moderate to Vigorous PA) | 1.6  (0.2, 3.4) | 1.7  (0.4, 4.7) | 1.6  (0.6, 4.3) | 0.8  (0.1, 3.1) | 1.9  (0.6, 4.8) | 1.3  (0.1, 3.6) | 1.2 [-0.3, 2.7] | 0.13 | -0.6 [-1.7, 0.5] | 0.31 |
| Actual Time <1.5 METs (mins/ day) *  (Sedentary Behaviour) | 1114.6 (1018.1, 1212.5) | 1108.8 (997.9, 1202.4) | 1129 (1013.8, 1226.9) | 1137.6 (1006.6, 1258.6) | 1044 (934.6, 1146.2) | 1118.9 (983.5, 1206.7) | -70.6 [-116.6, -24.5] | 0.002 | -13 [-59, 31.7] | 0.57 |
| Actual Time 1.5-3 METs (mins/ day) *  (Light Physical Activity) | 280.8 (201.6, 380.2) | 288 (203, 394.6) | 275 (198.7, 373) | 267.8 (168.5, 401.8) | 335.5 (267.8, 446.4) | 299.5 (204.5, 393.1) | 53.3 [13, 93.6] | 0.009 | 21.6 [-18.7, 60.5] | 0.3 |
| Actual Time > 3 METs (mins/ day) *  (Moderate to Vigorous PA) | 23  (2.9, 49) | 24.5  (5.8, 67.7) | 23  (8.6, 61.9) | 11.5  (1.4, 44.6) | 27.4  (8.6, 69.1) | 18.7  (1.4, 51.8) | 17.3 [-4.3, 38.9] | 0.13 | -8.6 [-24.5, 7.2] | 0.31 |
| **Physical Activity on Tue, Thur, Sat, Sun (Non-Exercise Intervention Days):** | | | | | | |  |  |  |  |
| N | 177 | 175 | 169 | 130 | 130 | 130 |  |  |  |  |
| PAEE  (kj^-1^ kg^-1^ day^-1^) * | 29.7  (22, 39.5) | 30.2  (23.3, 42.3) | 30.3  (20.5, 43) | 28.9  (19.8, 38.5) | 27.2  (19.8, 42.2) | 28  (21.4, 44.7) | -1.7 [-6.2, 2.8] | 0.47 | -1.1 [-6.3, 4.1] | 0.68 |
| PAEE (Accelerometry Only) *  (kj^-1^ kg^-1^ day^-1^) | 24.5  (16.8, 29.7) | 23.8  (16.3, 30.5) | 20  (12.7, 28) | 22.9  (14, 30.8) | 21.3  (14.9, 31.6) | 19.5  (14.4, 27.7) | -0.2 [-3.7, 3.3] | 0.91 | 0.6 [-3.2, 4.3] | 0.77 |
| Percentage Time < 1.5 METs (%) *  (Sedentary Behaviour) | 78.6  (69.9, 84.4) | 76.5  (71, 82.6) | 77.7  (71.4, 85.4) | 79.6  (71.5, 86) | 79  (69, 85.8) | 77.1  (69.6, 83.7) | 0.2 [-2.5, 2.9] | 0.88 | -1.5 [-4.6, 1.6] | 0.34 |
| Percentage Time 1.5 to 3 METs (%) *  (Light Physical Activity) | 18.8  (13.4, 27.4) | 21.3  (15.8, 25.3) | 18.3  (13.1, 24.6) | 18.1  (12.6, 25.8) | 19.5  (13.3, 26.9) | 20.1  (14.2, 26.3) | 0.0 [-2.4, 2.4] | 0.99 | 2.6 [-0.1, 5.2] | 0.054 |
| Percentage Time > 3 METs (%) *  (Moderate to Vigorous PA) | 1  (0.4, 3.1) | 1.2  (0.1, 3.3) | 1.9  (0.5, 4.7) | 1.1  (0.1, 3.4) | 1.1  (0.1, 3.1) | 1.2  (0.3, 3.5) | -0.2 [-1.3, 0.8] | 0.65 | -1.1 [-2.2, 0.0] | 0.054 |
| Actual Time <1.5 METs (mins/ day) *  (Sedentary Behaviour) | 1131.8  (1006.6, 1215.4) | 1101.6  (1022.4, 1189.4) | 1118.9  (1028.2, 1229.8) | 1146.2  (1029.6, 1238.4) | 1137.6  (993.6, 1235.5) | 1110.2  (1002.2, 1205.3) | 2.9 [-36, 41.8] | 0.88 | -21.6 (-66.2, 23) | 0.34 |
| Actual Time 1.5-3 METs (mins/ day) *  (Light Physical Activity) | 270.7  (193, 394.6) | 306.7  (227.5, 364.3) | 263.5  (188.6, 354.2) | 260.6  (181.4, 371.5) | 280.8  (191.5, 387.4) | 289.4  (204.5, 378.7) | 0.3 [-34.6, 34.6] | 0.99 | 37.4 (-1.4, 74.9) | 0.054 |
| Actual Time > 3 METs (mins/ day) *  (Moderate to Vigorous PA) | 14.4  (5.8, 44.6) | 17.3  (1.4, 47.5) | 27.4  (7.2, 67.7) | 15.8  (1.4, 49) | 15.8  (1.4, 44.6) | 17.3  (4.3, 50.4) | -2.9 [-18.7, 11.5] | 0.65 | -15.8 [-31.7, 0.1] | 0.054 |

MET: Metabolic Equivalent of Task.

PAEE: Physical Activity Energy Expenditure.

*Denotes variables with a skewed distribution, in which case the median and (interquartile range) are presented. Otherwise, data are presented as means ± standard deviations.

**Refers to the difference in the change in the outcome during the intervention in the exercise group participants who attended 85% of the scheduled exercise sessions, compared to the control group, using linear regression with the outcome variable being the mid-intervention follow-up measure at week six, and the exposure variable being the randomisation group, adjusted for the baseline measure of the outcome.

***Refers to the difference in the change in the outcome after the intervention in the exercise group participants who attended 85% of the scheduled exercise sessions, compared to the control group, using linear regression with the outcome variable being the post-intervention follow-up measure, and the exposure variable being the randomisation group, adjusted for the baseline measure of the outcome.
